# miRNA-Induced Downregulation of IPMK in Macrophages Mediates Lipopolysaccharide-Triggered TLR4 Signaling

**DOI:** 10.3390/biom13020332

**Published:** 2023-02-09

**Authors:** Haein Lee, Eunha Kim, Seyun Kim

**Affiliations:** 1 Department of Biological Sciences, Korea Advanced Institute of Science and Technology (KAIST), Daejeon 34141, Republic of Korea; 2 KAIST Institute for the BioCentury, KAIST, Daejeon 34141, Republic of Korea; 3 KAIST Stem Cell Center, KAIST, Daejeon 34141, Republic of Korea

**Keywords:** IPMK, macrophage, toll-like receptor, inflammation, TRAF6

## Abstract

Inositol polyphosphate multikinase (IPMK) is a pleiotropic enzyme responsible for the production of inositol polyphosphates and phosphoinositide. IPMK in macrophages was identified as a key factor for the full activation of the Toll-like receptor 4 (TLR4) signaling pathway and inflammation by directly interacting with tumor necrosis factor receptor-associated factor 6 (TRAF6). Here, dynamic changes of IPMK levels in lipopolysaccharide (LPS)-stimulated macrophages and their functional significance were investigated. Both the mRNA and protein levels of IPMK were acutely decreased in mouse and human macrophages when cells were stimulated with LPS for between 1 and 6 h. Analysis of the 3’ untranslated region (UTR) of mouse IPMK mRNA revealed a highly conserved binding site for miR-181c. Transfection of miR-181c mimics into RAW 264.7 macrophages led to decreased IPMK 3’UTR-luciferase reporter activity and lowered endogenous IPMK levels. When the genomic deletion of a 33-bp fragment containing a putative miR-181c-binding site was introduced within the IPMK 3’UTR of RAW 264.7 macrophages (264.7^Δ3′UTR^), LPS-triggered downregulation of IPMK levels was prevented. LPS treatment in 264.7^Δ3′UTR^ macrophages decreased TLR4-induced signaling and the expression of proinflammatory cytokines. In response to LPS stimulation, K63-linked ubiquitination of TRAF6 was impaired in 264.7^Δ3′UTR^ macrophages, suggesting an action of IPMK in the suppression of TRAF6 activation. Therefore, our findings reveal that LPS-mediated suppression of IPMK regulates the full activation of TLR4 signaling and inflammation in macrophages.

## 1. Introduction

Inositol polyphosphate multikinase (IPMK) is a versatile signaling factor showing pleiotropic enzymatic activities that catalyze the synthesis of inositol polyphosphates such as inositol 1,3,4,5,6-pentakisphosphate as well as phosphatidylinositol 3,4,5-triphosphates [1,2,3]. These downstream products of IPMK are known to control downstream signaling effectors, such as Akt kinase, Btk kinase, and histone deacetylase [4,5,6,7]. In addition to its catalytic action, IPMK noncatalytically regulates diverse cellular activities by directly interacting with prime signaling factors including mechanistic target of rapamycin, serum response factor, and p53 [8,9,10,11]. Thus, IPMK acts as a multifaceted signaling hub in mammalian cells to coordinate the activity of various signaling networks. Our previous study further expanded our view on the function of IPMK by demonstrating that IPMK in macrophages is critical for the regulation of TLR4-mediated innate immune signaling activation [12].

Microbe-sensing TLRs are essential for host defense against invading pathogens. In immune cells, such as macrophages, activation of TLR4 stimulates inflammatory signaling pathways that ultimately lead to the robust synthesis of proinflammatory cytokines (e.g., interleukin-6) [13]. Recognition of TLR4 with specific pathogen-associated molecules initiates a series of signal transduction pathways by recruiting MyD88 (myeloid differentiation primary response 88) adaptor proteins [14]. The TLR4/MyD88 complex recruits interleukin-1 (IL-1) receptor-associated kinases (IRAKs), subsequently interacting with tumor necrosis factor receptor-associated factor 6 (TRAF6), an E3 ligase, to trigger lysine 63 (K63) auto-polyubiquitination of TRAF6 [15,16,17]. Polyubiquitinated active TRAF6 forms a recognition signal for the recruitment of TGF-β-activated kinase (TAK) 1 binding protein (TAB) 2/3 to activate TAK1 [18,19,20,21]. These molecular interactions are critical for the activation of downstream signaling effectors, such as c-Jun N-terminal kinase (JNK) and nuclear factor κB (NF-κB), as well as for the transcriptional activation of proinflammatory cytokines [22,23,24,25]. Dysregulation of TLR4 signaling and inflammatory responses can thus lead to pathological conditions (e.g., sepsis) and tissue damage [26].

MicroRNAs (miRNAs) are small non-coding RNAs, which regulate gene expression by generally pairing with complementary sequences within the untranslated regions (UTR) of target mRNAs, thereby controlling their stability and/or translation [27]. The endogenously expressed miRNAs have been discovered as critical contributors to many biological processes, such as immunity [28,29]. Accumulating studies have demonstrated that miRNAs are key regulators of macrophage activation and polarization [30,31]. In response to TLR ligands, several miRNAs have been shown to directly target components of the TLR signaling system, suggesting the significance of miRNAs in feedback regulatory mechanisms. For instance, miR-146a targets TRAF6 and IRAK1, key adaptor proteins of the TLR signaling cascade essential for NF-κB activation [32]. miR-23a was also shown to promote macrophage polarization to the M2 phenotype by directly suppressing TLR and interferon (IFN) signaling [33]. miR-27-3p regulates pro-inflammatory cytokine production in alveolar macrophages by targeting peroxisome proliferator-activated receptor-γ (PPAR-γ) [34]. miR-211 was also found to promote polarization of M1 macrophages via suppressor of cytokine signaling1 (SOCS1) [35]. LPS-stimulated Akt1 signaling in macrophages appear to govern levels of miRNAs (e.g., let-7e, miR-181c, miR-155, miR-125b) to control endotoxin sensitivity and tolerance via modulating TLR4 and SOCS1 [36].

Recent studies have highlighted the importance of IPMK in macrophages [12]. Conditional deletion of myeloid IPMK in mice decreases the production of proinflammatory cytokines and protects mice from excessive immune responses such as polymicrobial septic shock, underscoring the physiological role of macrophage IPMK in innate immunity. The direct interaction between IPMK and TRAF6 appears to be critical, as IPMK can prevent the proteasomal degradation of TRAF6. When macrophages are stimulated with LPS, the interaction between TRAF6 and IPMK becomes disrupted and TRAF6 binds to upstream signaling activators such as IRAK1. The loss of IPMK in macrophages thus increases TRAF6 K48 ubiquitination and promotes its degradation, thereby limiting the activation of TLR4 signaling. Consequently, conditional deletion of IPMK in myeloid cells protects mice against polymicrobial sepsis and lipopolysaccharide-induced systemic inflammation.

To the best of our knowledge, no previous studies have determined whether IPMK in macrophages undergoes dynamic expression control. Moreover, the detailed molecular mechanisms through which IPMK modulates macrophage functions remain poorly understood. The present study provides evidence supporting that IPMK expression in macrophages is transcriptionally suppressed under LPS stimulation. In mutant macrophages in which IPMK expression is not sensitive to LPS treatment, TLR4 signaling and proinflammatory cytokine production were attenuated, which was accompanied by reduced K63-linked ubiquitination of TRAF6. Collectively, our findings demonstrate that suppression of IPMK expression is a critical event required for the full and sustained activation of innate immune signaling and inflammatory response in LPS-stimulated macrophages, suggesting that modulation of macrophage IPMK levels could be valuable for the management of uncontrolled inflammatory diseases such as sepsis.

## 2. Materials and Methods

### 2.1. Cell Culture and Cell Line Generation

Reagents used for cell culture are as follows: high-glucose Dulbecco’s modified Eagle’s medium (DMEM) (WELGENE, Inc., Gyeongsan, Republic of Korea), RPMI 1640 medium (WELGENE, Inc., Gyeongsan, Republic of Korea), Dulbecco’s phosphate-buffered saline (DPBS) (WELGENE, Inc., Gyeongsan, Republic of Korea), penicillin/streptomycin (WELGENE, Inc., Gyeongsan, Republic of Korea), 0.25% trypsin-EDTA (WELGENE, Inc., Gyeongsan, Republic of Korea), fetal bovine serum (FBS) (Atlas Biologicals, Inc., Fort Collins, CO, USA and Thermo Fisher Scientific, Waltham, MA, USA), sodium pyruvate (Invitrogen, Waltham, MA, USA), HEPES (Invitrogen, Waltham, MA, USA), recombinant mouse M-CSF (R&D Systems, Minneapolis, MN, USA), and LPS (Sigma-Aldrich, St. Louis, MO, USA).

Isolation of BMDMs was performed as described previously [37,38]. Adherent BMDMs were detached on day six and plated in six-well culture plates. RAW 264.7 and HEK293T cells were grown in high-glucose DMEM supplemented with 10% FBS, 2 mM L-glutamine, and 100 µg/mL penicillin/streptomycin. Lipofectamine LTX was used for the introduction of microRNA into RAW 264.7 cells, as described by the manufacturer (Invitrogen, Waltham, MA, USA).

To generate a cell line in which the putative miR-181c binding sequence was deleted from the 3’UTR of IPMK, CRISPR/Cas9 editing system was used. We designed two guide RNAs targeting putative miR-181c binding sequence using Cas-Designer (Available online: http://www.rgenome.net/cas-designer/ (accessed on 2 February 2023)) [39,40]. RAW 264.7 cells were seeded at 5 × 10^5^ cells in a six-well cell culture plate and incubated for 24 h at 37 °C and 5% CO_2_. After incubation, two guide RNA constructs and a Cas9-GFP expressing plasmid were co-transfected with FuGENE^®^ HD Transfection Reagent (E2311, Promega, Madison, WI, USA). After 24 h of transfection, GFP-positive cells were sorted into a 96-well plate by a single cell using MoFlo Astrios EQ cell sorter (Beckman Coulter, Brea, CA, USA). Cells were gradually transferred to larger plates when they reached 70% confluency. The exact location of the deletion site was confirmed by sequencing.

### 2.2. Immunoblotting and Immunoprecipitation

For immunoblot analyses, cells were washed twice with PBS and lysed in lysis buffer consisting of 1% NP-40, 120 mM NaCl, 40 mM pH 7.4 Tris-HCl, 1 mM ethylenediaminetetraacetic acid (EDTA), 1.5 mM sodium orthovanadate, 50 mM sodium fluoride, 10 mM sodium pyrophosphate, and protease inhibitor cocktail (Roche, Basel, Switzerland). Protein concentrations were determined by Bradford Protein Assay (Bio-Rad Laboratories, Hercules, CA, USA). Immunoprecipitation was performed as described previously [12].

Antibodies against the following proteins were purchased from the indicated sources: phospho-IKKα/β (2697), IKKα (2682), IKKβ (2370), phospho–NF-κB (3033), NF-κB (8242), phospho-IκB (2859), IκB (4814), phospho-JNK (4668), and K63-linkage Specific Polyubiquitin (5621) (Cell Signaling Technology, Danvers, MA, USA); JNK1/2 (554285; BD Biosciences, Franklin Lakes, NJ, USA); TRAF6 (sc-7221), GAPDH (sc-32233) and HSP90 (sc-13119) (Santa Cruz Biotechnology, Dallas, TX, USA); Rabbit IgG isotype control (02-6102) (Invitrogen, Waltham, MA, USA); Tubulin (T5109) (Sigma-Aldrich, St. Louis, MO, USA); IPMK (rabbit polyclonal antibody, raised against a mouse IPMK peptide corresponding to amino acids 295-311 (SKAYSTHTKLYAKKHQS; Covance) containing an added N-terminal cysteine) [8].

### 2.3. RNA Isolation and RT-qPCR

Total RNA was isolated from cells using Tri reagent (Molecular Research Center, Inc., Cincinnati, OH, USA) according to the manufacturer’s protocol. 1 µg of total RNA was used for the synthesis of first-strand cDNA by reverse transcriptase. Quantitative real-time PCR analysis was performed with the SYBR Green master mix (Toyobo, Osaka, Japan) and the Step One Plus Real-Time PCR system (Applied Biosystems, Waltham, MA, USA). The expression level of the genes of interest was normalized to the expression level of the *36b4* gene or *Hnrnpab* gene as the housekeeping control and presented as fold changes over baseline using the ΔΔCt method. The cycling program was 10 min at 95 °C, followed by 40 cycles of 95 °C for 15 s, 58 °C for 30 s, and 72 °C for 30 s. A melting curve was determined after the completion of qPCR. Primer sequences for qPCR are as follows: 36b4 (forward: 5′-TCACTGTGCCAGCTCAGAAC-3′; reverse: 5′-AATTTCAATGGTGCCTCTGG-3′), Hnrnpab (forward: 5′-GGGAGGTCTAAACCCTGAAG-3′; reverse: 5′-GGGCAACCTTGATTTCACAC-3′), Ipmk (forward: 5′-5′-CCAAAATATTATGGCATCTG-3′; reverse: 5′-TATCTTTACATCCATTATAC-3′), Il-1β (forward: 5′-GCCTCGTGCTGTCGGACC-3′; reverse: 5′-TGTCGTTGCTTGGTTCTCCTTG-3′), Il-6 (forward: 5′-ATGAACAACGATGATGCACTT-3′; reverse: 5′-TATCCAGTTTGGTAGCATCCAT-3′), Tnf-α (forward: 5′-CACAAGATGCTGGGACAGTGA-3′; reverse: 5′-GAGGCTCCAGTGAATTCGGA-3′).

### 2.4. Multiplex Immunoassay

After treatment of LPS to the 264.7^WT^ and 264.7^Δ3′UTR^ cells, the respective cell culture medium was collected. The level of IL-6 in the media was measured using Bio-Plex Pro Mouse Cytokine IL-6 (Bio-Rad Laboratories, Hercules, CA, USA) and analyzed using Bio-Plex 200 Systems (Bio-Rad Laboratories, Hercules, CA, USA) following the manufacturer’s instructions.

### 2.5. UTR Reporter Assays

The putative binding sites for miR-181c of 3′UTR of IPMK and the mutants were cloned into the Psicheck 2 dual-luciferase reporter vector (Promega, Madison, WI, USA). HEK293T cells co-transfected with each reporter construct and miR-181c mimic or mimic controls (Thermo Fisher Scientific, Waltham, MA, USA). Cells were lysed at 24 hours after transfection, and the ratio of Renilla to firefly luciferase was measured with the dual luciferase assay (Promega, Madison, WI, USA).

### 2.6. Statistical Analysis

Differences between averages of RT-qPCR and multiplex immunoassay were analyzed using a two-tailed Student’s t test, one-way ANOVA followed by Tukey’s post hoc test, two-way ANOVA followed by Tukey’s post hoc test, or the Mann-Whitney test. Each statistical method was specified in the legend. Statistical significance was set at *p* < 0.05. Statistical calculations were performed in Microsoft Excel for a two-tailed Student’s t test and GraphPad Prism 7 software for a one-way, two-way ANOVA followed by Tukey’s post hoc test and a Mann-Whitney test. All data were expressed as means ± SE.

## 3. Results

### 3.1. Expression of Macrophage IPMK Is Dynamically Regulated by LPS

**Figure 1:**
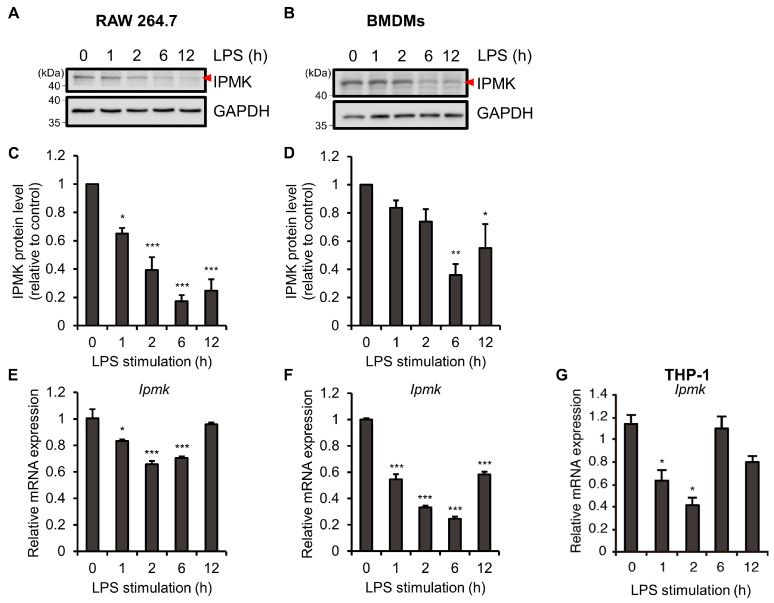
LPS treatment dynamically regulates IPMK expression levels in macrophages. (**A**–**G**) Macrophages were stimulated with LPS (100 ng/mL) for the indicated times. Levels of IPMK protein (red triangle) were analyzed by immunoblotting lysates of (**A**) RAW 264.7 cells and (**B**) BMDMs. Quantification of IPMK protein levels obtained from at least three independent experiments. Densitometric data were normalized to GAPDH control for (**C**) RAW 264.7 cells (*n* = 4) and (**D**) BMDMs (*n* = 3). Bars represent means ± SE. (**E**–**G**) mRNA levels of *Ipmk* were quantified by RT-qPCR in (**E**) RAW 264.7 cells, (**F**) BMDMs, and (**G**) THP-1 cells. Bars represent means ± SE (*n* = 3). * *p* < 0.05, ** *p* < 0.01, *** *p* < 0.001 (one-way ANOVA followed by Tukey’s post hoc test (**C**–**G**)).

As a first step to characterize the changes of IPMK in TLR4 signaling, we examined whether IPMK protein levels were affected by LPS (i.e., a TLR4 ligand). As shown in Figure 1A, IPMK protein levels were reduced in murine macrophage RAW 264.7 cells with as little as 2 h of LPS exposure. At 6 h of LPS treatment, the IPMK protein levels were markedly decreased compared to untreated cells (Figure 1A,C). Similar results were obtained using mouse bone-marrow-derived macrophages (BMDMs) (Figure 1B,D). To test whether this dynamic change in IPMK protein levels reflects transcriptional control, the mRNA levels of IPMK were analyzed. Similar to protein changes, the IPMK mRNA levels in macrophages were acutely decreased during the initial 1–2 h of LPS treatment, but gradually returned to the original level at later time points (Figure 1E,F), as well as human THP-1 macrophages (Figure 1G). These results demonstrated that acute suppression of IPMK gene expression in macrophages occurs at the transcriptional level in response to LPS.

### 3.2. Generation of Mutant Macrophages in Which IPMK Expression Is Not Sensitive to LPS

**Figure 2:**
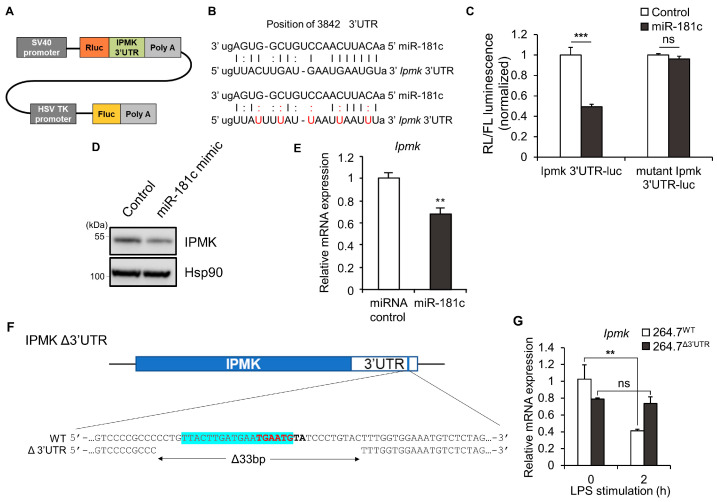
33-bp deletion of IPMK 3′UTR attenuates the TLR4 signaling response by LPS. (**A**) Schematic representation of the reporter plasmid psiCheck2 IPMK 3′UTR. (**B**) Predicted miR-181c binding site in the IPMK 3′UTR. Perfect matches are indicated by vertical lines; G:U pairs by colons. Red letters represent point mutations introduced to disrupt miRNA binding. (**C**,**D**) Relative luciferase activities were measured in HEK293T cells 24 h after co-transfection with a luciferase reporter plasmid and miR-181c mimic (**C**). Levels of endogenous IPMK protein were analyzed by immunoblotting the same lysates (**D**,**E**) *Ipmk* mRNA expression levels in RAW 264.7 cells were measured by RT-qPCR. (**F**) Schematic diagram showing the IPMK 3’UTR deletion of the putative miR-181c binding site which is highlighted in blue. The seed sequence is labeled in bold. (**G**) Levels of *Ipmk* mRNA expression were measured by RT-qPCR in 264.7^WT^ and 264.7^Δ3′UTR^ cells after LPS (100 ng/mL) treatment for 2 h. Data shown are representative of three independent experiments and are presented as means ± SE (*n* = 3). ** *p* < 0.01, *** *p* < 0.001, ns; not significant (two-way ANOVA followed by Tukey’s post hoc test (**C**,**G**), Student’s *t* test (**E**)).

We next focused on 3′ untranslated regions (3′ UTRs), which have been shown to act as a post-transcriptional regulatory element [41]. In many cases, micro RNAs (miRNAs) interact with the 3′ UTRs of target mRNAs to induce mRNA degradation and translational repression [42,43]. We searched for predicted binding sites for mouse miRNAs within the 3’UTR of the mouse IPMK gene using the TargetScan [44,45] and miRDB [46,47], and identified several putative sites for miRNAs. Among them, miRNA-181c stood out due to its known ability to suppress TLR signaling in activated macrophages [36]. To confirm that miR-181c targets the IPMK 3′UTR, a reporter assay was performed using a vector harboring the putative miR-181c binding site from the IPMK 3′UTR downstream of the luciferase gene (IPMK-3′UTR-luc) (Figure 2A–C). While miR-181c overexpression in HEK293T cells reduced the luciferase activity associated with the wild-type (WT) IPMK 3′UTR-luc by 60%, it did not affect the luciferase activity associated with an IPMK 3′UTR carrying mutations designed to interfere with the binding of the miR-181c seed sequence (mut IPMK-3′UTR-luc) (Figure 2C). Transfection of miR-181c significantly reduced endogenous IPMK protein levels when compared to transfection of a scrambled miRNA control (Figure 2D). We further performed same experiment using RAW 264.7 cells and observed that *Ipmk* mRNA levels were reduced in cells overexpressing miRNA-181c mimic (Figure 2E).

To further test the effects of this 3′UTR site, mutant RAW 264.7 cell lines harboring a genomic deletion in the 3′UTR of IPMK were generated using the CRISPR/Cas9 editing system. The correct deletion of the 33-bp fragment was followed and validated by sequencing of amplified genomic regions from single cell-derived colonies (Figure 2F). Compared to wild-type cells (264.7^WT^), LPS-stimulated reduction of *Ipmk* mRNA levels was resistant in IPMK 3′UTR-deletion macrophages (264.7^Δ3′UTR^). After 2 h of LPS treatment, *Ipmk* mRNA levels were protected in 264.7^Δ3′UTR^ compared to 264.7^WT^ cells, suggesting that LPS-triggered downregulation of IPMK can be prevented by the genomic editing of IPMK 3′UTR (Figure 2G).

### 3.3. TLR4 Signaling and Cytokine Expression Are Reduced in LPS-Stimulated 264.7^Δ3′UTR^ Macrophages

**Figure 3:**
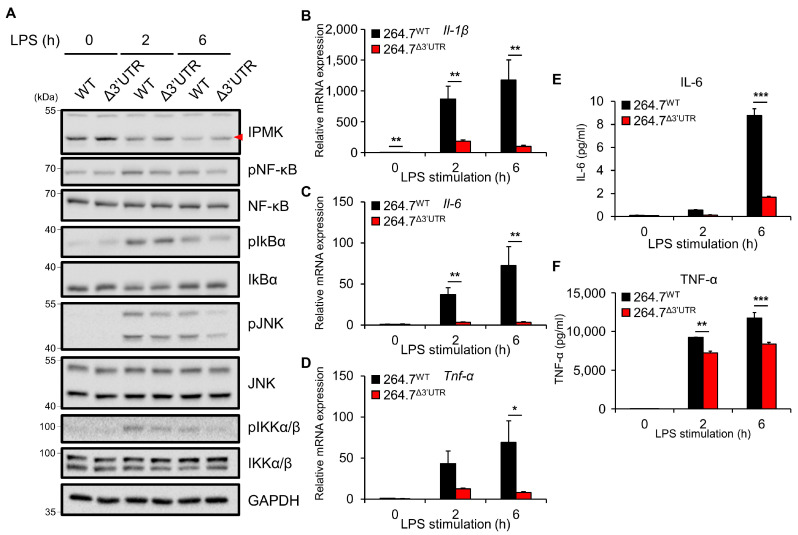
264.7^Δ3′UTR^ cells exhibit downregulated TLR4-dependent inflammatory responses. (**A**) Phosphorylation of TLR4 downstream signaling molecules was analyzed by immunoblotting lysates of 264.7^WT^ or 264.7^Δ3′UTR^ cells stimulated for 2 h or 6 h with 100 ng/mL of LPS. The red triangle indicates the target band. (**B**–**D**) mRNA levels of proinflammatory cytokines *Il-1β* (**B**), *Il-6* (**C**) and, TNF-α (**D**) were measured by RT-qPCR in 264.7^WT^ or 264.7^Δ3′UTR^ cells 0, 2, or 6 h after stimulation with 100 ng/mL of LPS. (**E**,**F**) Secreted levels of the IL-6 (**E**) and TNF-α (**F**) in culture medium were measured by multiplex immunoassay after stimulation with LPS (100 ng/mL). Data shown are representative of at least three independent experiments and are presented as means ± SE. * *p* < 0.05, ** *p* < 0.01, *** *p* < 0.001 (Mann-Whitney test (*n* = 6, **B**–**D**), two-way ANOVA followed by Tukey’s post hoc test (*n* = 3, **E**,**F**)).

Using 264.7^Δ3′UTR^ cells, we next addressed the functional significance of IPMK in LPS-activated conditions. As expected, downregulation of IPMK protein levels in response to 2 or 6 h of LPS treatment was blunted in 264.7^Δ3′UTR^, relative to 264.7^WT^ cells (Figure 3A). The inflammatory responses of 264.7^Δ3′UTR^ cells were analyzed by measuring TLR4 signaling and proinflammatory cytokine production in response to LPS. At 2 h of an early and acute phase of LPS stimulation, 264.7^Δ3′UTR^ macrophages exhibited reduced activation of NF-κB and JNK signaling pathways, compared to 264.7^WT^ (Figure 3A). Moreover, at 6 h of LPS treatment, this signaling defect was more profound, with significantly lower phosphorylation of NF-κB, JNK, and IKKα/β in 264.7^Δ3′UTR^ cells (Figure 3A and Supplementary Figure S1). Notably, unlike 264.7^WT^ control cells in which *Il-1β* and *Il-6* were robustly induced, the cytokine mRNA levels from 264.7^Δ3′UTR^ cells were markedly decreased after 2 h of LPS treatment (Figure 3B–D). We further found that secreted protein levels of the proinflammatory cytokines IL-6 and TNF-α were significantly reduced in LPS-stimulated 264.7^Δ3′UTR^ cells compared with those from 264.7^WT^ control cells (Figure 3E,F). Taken together, these results suggest that the downregulation of IPMK in LPS-stimulated macrophages is a critical for the full activation of TLR4 signaling transmission as well as associated cytokine induction responses.

### 3.4. The Activation of TRAF6 Was Decreased in LPS-Stimulated 264.7^Δ3′UTR^ Macrophages

**Figure 4:**
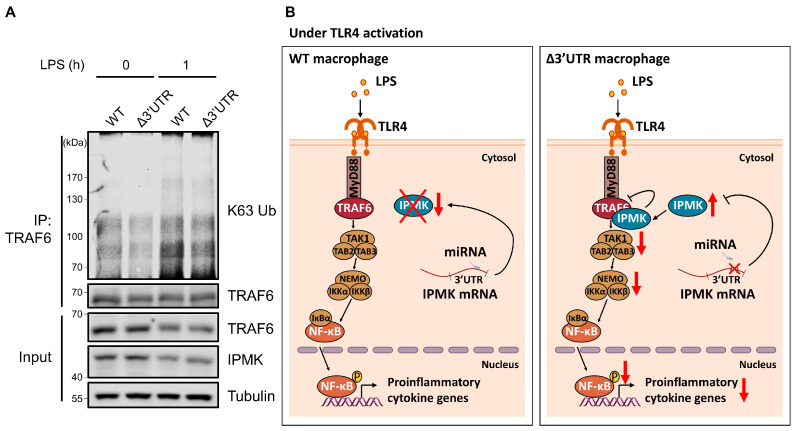
K63-linked ubiquitination of TRAF6 was decreased in IPMK Δ3’UTR macrophages. (**A**) Levels of endogenous TRAF6 K63 ubiquitination were measured in either 264.7^WT^ or 264.7^Δ3′UTR^ cells in the absence or presence of LPS (100 ng/mL) treatment for 1 h. Cell lysates were subject to immunoprecipitation with anti-TRAF6 antibodies, followed by immunoblot analysis with anti-K63 ubiquitin-specific antibodies. (**B**) Model depicting the regulation of TLR4 signaling by IPMK. In LPS-stimulated macrophages, *Ipmk* mRNA and protein levels were decreased, thus allowing the full transmission of TLR4-triggered signaling and inflammatory gene expression. When this acute downregulation of IPMK is not properly working by the deletion of *Ipmk* 3′UTR, K63-linked ubiquitination of TRAF6 becomes lowered, which impairs the activation of TLR4 signaling and inflammatory response.

In 264.7^Δ3′UTR^ cells, downstream effectors such as JNK and NF-κB were markedly reduced and their IKK phosphorylation was also decreased, suggesting a signaling defect at the upstream level. Previously, IPMK was found to directly bind to TRAF6 and protect its degradation in resting macrophages [12]. However, previous studies have not explored whether IPMK could influence TRAF6 activation through K63-linked ubiquitination in LPS-activated macrophages. To test this possibility, we performed endogenous TRAF6 immunoprecipitation and immunoblotting of endogenous K63 ubiquitination in LPS-stimulated 264.7^Δ3′UTR^. TRAF6 K63 ubiquitination was significantly lowered in 264.7^Δ3′UTR^ cells compared to the control cells (Figure 4A), indicating that IPMK in 264.7^Δ3′UTR^ cells can interfere with the full activation of TRAF6. These findings suggest that rapid and acute downregulation of IPMK in macrophages upon LPS stimulation is critical to enable the activation of TRAF6 and its downstream TLR4 signaling events and elicit a proper inflammatory response (Figure 4B).

## 4. Discussion

Despite the fundamental importance of IPMK in various cellular signaling events, there are still several unsolved questions regarding the functional significance of IPMK in the control of macrophage functions. The present study provides several lines of evidence that demonstrate that IPMK is a key molecular target involved in the fine-tuning of TLR4 signaling in macrophages. (i) IPMK expression in macrophages is acutely downregulated at both the mRNA and protein levels in response to LPS stimulation. This reduced expression becomes restored at later stages of LPS treatment. (ii) IPMK appears to be a target for miRNAs, as miRNA-181c, a putative miRNA, successfully depleted IPMK via its 3′UTR. (iii) Introducing a genomic deletion within the *Ipmk* 3′UTR of RAW 264.7 cells (264.7^Δ3′UTR^) abolishes the LPS-stimulated suppression of IPMK, thereby maintaining higher levels of IPMK compared to the control cells. (iv) In response to LPS, TLR4 signaling and proinflammatory cytokine expression are reduced in 264.7^Δ3′UTR^ cells. (v) K63-linked ubiquitination of TRAF6 was selectively reduced in LPS-stimulated 264.7^Δ3′UTR^ macrophages.

Importantly, to the best of our knowledge, our study is the first to report that the level of IPMK is dynamically regulated by an external stimulus. Our findings strongly suggest that miRNA could be the main driver of IPMK expression control. miRNA-181c appeared to be a promising candidate and treatment of miRNA-181c mimic in our experimental conditions reduced endogenous IPMK levels, but failed to achieve complete depletion, suggesting that different miRNAs can work together to control IPMK mRNA levels. Two previous studies reported that human IPMK expression can be targeted by miRNA [48,49]. A study in epithelial ovarian cancer suggested that IPMK is one of the targets of miR-18a, thereby mediating cancer cell growth [48]. MiR-23b was also found to directly target IPMK and negatively regulate autophagy activity, which modulates the Akt/mTOR/autophagy pathway, as well as inflammation [49]. In addition to identifying more miRNAs targeting IPMK 5′ and 3′UTRs to elucidate miRNA networks for controlling macrophage sensitivity and tolerance to LPS, transcriptional activation or repression of the IPMK gene should be further investigated. Long-term effects of LPS stimulation on IPMK expression in macrophages also remain to be investigated.

The functional significance of LPS-induced IPMK suppression uncovered in this study suggests that IPMK plays a significant role in controlling the activation of the TLR4 signaling pathway. However, the involvement of IPMK in mediating TLR4 signaling was much more complex than we initially expected. Particularly, optimal levels of IPMK in macrophages are required to stabilize TRAF6. When cells are stimulated by LPS and invading pathogens, TRAF6 dissociates from IPMK and engages with its signaling partners, such as IRAK. According to our findings, LPS-activated macrophages activate molecular systems (e.g., miRNAs) to efficiently downregulate IPMK levels. Given that IPMK can potentially interact with TRAF6, sustained IPMK expression could interfere with the inflammatory action of TRAF6. As shown in our study, IPMK could prevent TRAF6 activation in 264.7^Δ3′UTR^ cells, further dampening TRL4 signaling, as well as inflammatory cytokine expression. We thus speculated that IPMK levels and functions are tightly controlled in both resting and LPS-stimulated macrophages to elicit highly efficient immune responses. It would also be interesting to test whether this dynamic control of IPMK expression and function is effective in other cell types, such as osteocytes [50].

Recently, the identification of single-nucleotide polymorphisms (SNPs) in human IPMK from immune-mediated diseases (e.g., rheumatoid arthritis, psoriasis, and Crohn’s disease) suggests that IPMK contributes to the onset and progression of these diseases [51]. Given the importance of macrophages in immunity, our study suggests that regulation of IPMK levels could benefit the treatment of infectious and autoimmune disorders. Findings in the current study further highlight the key contribution of macrophage IPMK to the tight control of septic response. As revealed from our previous report, genetic deletion of myeloid IPMK in mice was shown to protect host from sepsis as well as LPS-stimulated inflammation. Since the main signaling action of macrophage IPMK appears to rely on its non-catalytic role in controlling TRAF6, developing ways to selectively deplete macrophage IPMK levels could be useful to manage uncontrolled inflammatory response, such as sepsis.

## Supplementary Materials

The following supporting information can be downloaded at: https://www.mdpi.com/article/10.3390/biom13020332/s1, Figure S1: 264.7^Δ3’UTR^ cells exhibit downregulated TLR4-dependent inflammatory responses.
