# Supplementary material for: miRNA-Induced Downregulation of IPMK in Macrophages Mediates Lipopolysaccharide-Triggered TLR4 Signaling
Source: Biomolecules. 2023 Feb 9;13(2):332. doi: 10.3390/biom13020332 (PMC9952907; doi:10.3390/biom13020332)
Supplement: Supplementary file 1 [file biomolecules-13-00332-s001.zip › biomolecules-2170952-supplementary.pdf]

biomolecules

MDPI

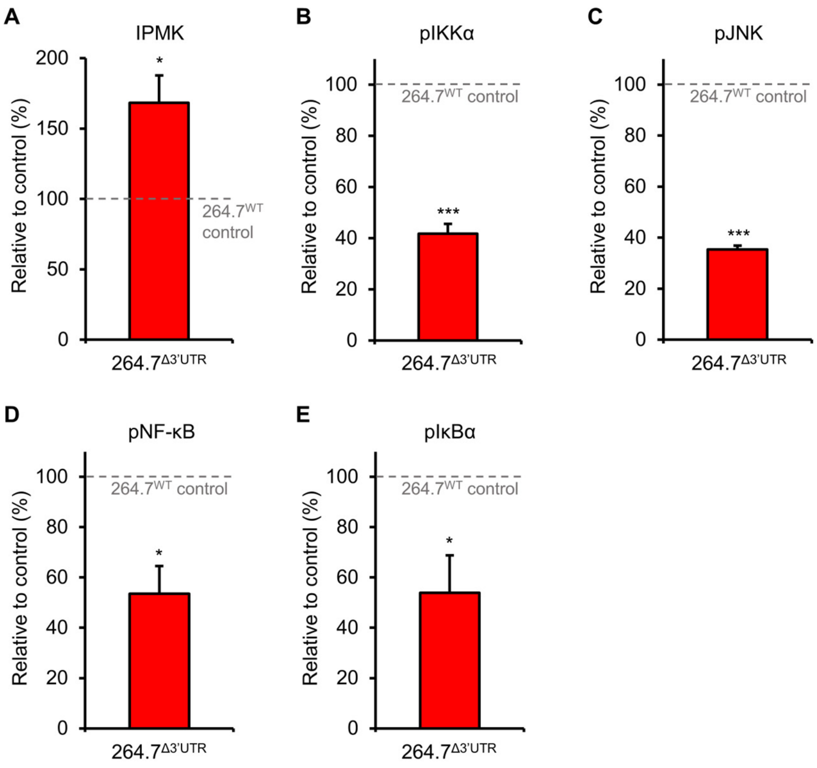

Figure S1. 264.7$^{\Delta 3'UTR}$ cells exhibit downregulated TLR4-dependent inflammatory responses. (A-E) Relative changes of IPMK and phosphorylated TLR4 signaling molecules in RAW 264.7 cells stimulated by 100 ng/mL LPS for 6 hours were quantitated. Densitometric results were normalized to GAPDH control. Bars represent mean ± SE (n = 3). *$p < 0.05$, ***$p < 0.001$ (two-tailed Student's t test).
